# Association of the Type of Public Pension With Mental Health Among South Korean Older Adults: Longitudinal Observational Study

**DOI:** 10.2196/49129

**Published:** 2024-05-02

**Authors:** Seung Hoon Kim, Hyunkyu Kim, Sung Hoon Jeong, Eun-Cheol Park

**Affiliations:** 1 Department of Ophthalmology Soonchunhyang University Hospital Cheonan Soonchunhyang University College of Medicine Chenonan Republic of Korea; 2 Institute of Health Services Research Yonsei University Seoul Republic of Korea; 3 Department of Psychiatry Yonsei University College of Medicine Seoul Republic of Korea; 4 Department of Rehabilitation Medicine Seoul National University Hospital Seoul Republic of Korea; 5 National Traffic Injury Rehabilitation Research Institute National Traffic Injury Rehabilitation Hospital Yangpyeong Republic of Korea; 6 Department of Preventive Medicine Yonsei University College of Medicine Seoul Republic of Korea

**Keywords:** depression, retirement, contributory public pension, low-income household, public health, mental health, data, big data, retirement, longitudinal data, low income

## Abstract

**Background:**

As income and health are closely related, retirement is considered undesirable for health. Many studies have shown the association between pension and health, but no research has considered the association between contribution-based public pensions or their types and health.

**Objective:**

This study investigates the association between the type of contributory public pension and depressive symptoms among older adults.

**Methods:**

We analyzed the data of 4541 older adults who participated in the South Korea Welfare Panel Study (2014-2020). Depressive symptoms were measured using the 11-item Center for Epidemiologic Studies Depression scale. Public pensions in South Korea are classified into specific corporate pensions and national pensions. For subgroup analyses, pensioners were categorized according to the amount of pension received and the proportion of public pension over gross income. Analyses using generalized estimating equations were conducted for longitudinal data.

**Results:**

Individuals receiving public pension, regardless of the pension type, demonstrated significantly decreased depressive symptoms (national pension: β=–.734; *P*<.001; specific corporate pension: β=–.775; *P*=.02). For both pension types, the higher the amount of benefits, the lower were the depression scores. However, this association was absent for those who received the smaller amount among the specific corporate pensioners. In low-income households, the decrease in the depressive symptoms based on the amount of public pension benefits was greater (fourth quartile of national pension: β=–1.472; *P*<.001; second and third quartiles of specific corporate pension: β=–3.646; *P*<.001).

**Conclusions:**

Our study shows that contributory public pension is significantly associated with lower depressive symptoms, and this association is prominent in low-income households. Thus, contributory public pensions may be good income sources for improving the mental health of older adults after retirement.

## Introduction

Depression in older adults decreases successful aging, adversely affecting not only the health-related quality of life of individuals but also of families and communities [1]. Older adults are more likely to be exposed to depression because they experience a series of adversities such as death, retirement, and health problems [2,3]. In old age, depression is a risk factor for nonsuicidal mortality, and both major and minor depression have been associated with suicide [4].

The prevalence of depression in South Korea (hereafter referred to as Korea) was 2.09% in 2019, which was lower than that reported in Japan (2.10%) and in the United States (4.38%) [5]. However, the prevalence of depression tends to increase with age, reaching 13.9% in those in their 60s and 70s and 18.4% in those in their 80s or older [6]. The proportion of older adults with depression reached 14% in 2018, and Korea is expected to become a superaged society by 2025, when the older adult population reaches 20.3% of the total population in Korea [7]. Therefore, a better understanding of depression in old age is of paramount importance from a clinical and public health perspective.

Income and health are closely related; to some extent, the more money people make, the better is their health [8]. This makes retirement, where a certain income for a person ceases, undesirable for health. In other words, old age income is important for health and other aspects [9]. Low economic status often leads to adjustment disorders accompanied by a depressed mood [10]. In Korea, the disposable income of older adults is less than 70% of the economy-wide average, which is the lowest among the Organization for Economic Co-operation and Development countries [11]. Moreover, Korea has a relative poverty rate of 45% among adults older than 65 years, which is the highest among the Organization for Economic Co-operation and Development countries. Relative poverty is defined as an income below half the national median equivalized household income.

As in many countries, Korea’s retirement income security system has a multilayered structure. First, the National Basic Livelihood Security System and the noncontributory basic old age pension are positioned as safety nets for the low-income class. Second, public pension, which provides pension benefits based on the subscribers, plays the most important role in guaranteeing retirement income [12]. The types of public pensions vary depending on the country, but in Korea, they are divided into (1) specific corporate pension for special workers such as public officials, private school faculty members, and military personnel, and (2) national pension for the general public [13,14]. Lastly, individuals can prepare additional retirement income by choosing to subscribe to a private pension operated by a company rather than the government. Among these multilayered income security systems, public pensions play a role in dispersing the risk of poverty that may occur among citizens. Furthermore, they allow the entire society to bear the costs evenly, without the double burden of paying for other people’s retirement living expenses on income-earning people due to those who are guaranteed a minimum living by relying on the public assistance system.

The relationship between health and pension, which guarantees income for the aged population, is debatable [15-18]. Unlike noncontributory pensions, which are paid only if income requirements are met (even if there is no certain contribution), few studies have been conducted on how contribution-based pensions (guaranteed for their contributions) affect the mental health of older adults [19]. When public pensions are divided into national pensions and specific corporate pensions, as in Korea, and there is a large difference in the amount received based on the type of pension (assuming that the contribution is different), the association of depression may differ depending on the type and amount of public pension received.

We hypothesized that a contribution-based pension would help the mental health of the older population and that larger public pension benefits would be more beneficial to mental health. It was also assumed that as the characteristics of the 2 pillars of Korea’s public pension are different, the degree of impact on mental health will also be different. Therefore, this study examines whether public pensions are associated with depressive symptoms, depending on the type and amount of receipt, among adults older than 65 years.

## Methods

### Study Population and Data

Data from the Korea Welfare Panel Study (KoWePS) conducted by the Korea Institute for Health and Social Affairs and Seoul National University were analyzed in this study. The KoWePS was a large-scale, annual, longitudinal panel survey conducted from 2006 (wave 1) to 2020 (wave 15). We used samples extracted from wave 9 (2014) to wave 15 (2020), as the 11-item Center for Epidemiologic Studies-Depression (CESD-11) scale that measures depressive symptoms has been partially changed since wave 9 (2014). The study participants were also limited to those who were 65 years or older and who did not have a history of depression in wave 9 in order to evaluate the association between pension benefits and depressive symptoms. After excluding participants with missing data, including CESD-11 score (n=254), 4541 respondents were included in the final sample of the baseline study year.

### Depressive Symptoms

The primary outcome was depressive symptoms, which were evaluated based on the 11-item version of the CESD scale. The CESD-11 is a well-validated self-reported screening tool and is a shorter version of the original 20-item instrument [20]. It is designed to evaluate 11 depressive symptoms for 1 week on a 4-point scale (0-3). The respondents reported symptoms for each year of the study period. The total score was multiplied by 20/11 to match the standard CESD-20 score. A score of 16 or more is clinically defined as having depression [21]. In our sample, the Cronbach α for the internal consistency of CESD-11 was .88, indicating high internal reliability.

### Types of Public Pensions

**Figure 1:**
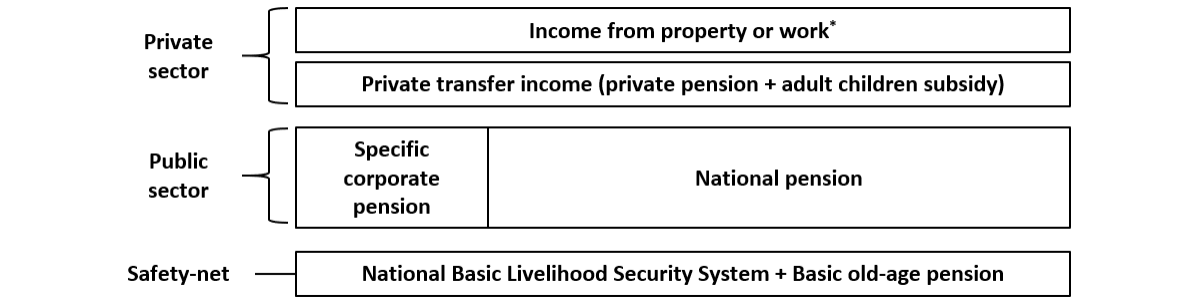
Schematic model of the Korean Retirement Income Security System. *Workers beyond the pension age.

Study participants were asked to report whether they received public pensions, the type of pension, and the annual pension benefit amount received at every wave of the KoWePS. The type of pension was divided into specific corporate pension for persons engaged in special occupations (eg, public officials, private school faculty members, military personnel, post office workers) and national pensions operated by the state for those ineligible individuals for special corporate pension but had certain income and paid contributions to the national pension system (Figure 1). For both types of public pensions in Korea, a person who has paid a certain percentage of the income for 10 years or more as a contribution can receive a monthly pension benefit when they reach a certain age between the ages of 60 and 65 years. However, the amount received depends on the contribution. Based on these categories, the independent variable was divided into 3 groups: no pension, national pension recipient, and specific corporate pension recipient. For the subgroup analyses, each pension recipient was classified according to the amount of pension benefits received per year. Finally, each public pension income was divided by the gross income to find the proportion of public pension income to the total income.

### Covariates

This study includes demographics, socioeconomic characteristics, health-related factors, and income-related factors as covariates, which reflected the results obtained for each wave. Demographic variables included sex, age, and region. Socioeconomic variables included marital status, number of household members, household income, highest level of education, and employment status. Health-related factors included smoking status, alcohol consumption, and chronic diseases. Income-related factors were realized property income, including interest or rental income; public transfer income, including basic old age pension, a noncontributory social pension in Korea; private transfer income, including income from the adult children and private pension; and household debt.

### Statistical Analyses

The distribution of general characteristics was calculated at baseline. Two-sided *t* tests, analysis of variance, and univariate linear regression were used to analyze the differences in the CESD-11 scores. The KoWePS data used in this study were hierarchically structured, and a stratified multistage probability design was used to select households, including multiple individuals from the same household [22]. For estimating the association between type of public pension and depressive symptoms, we used the generalized estimating equation (GEE) model, which can explain the time variations and correlations among the repeated measurements observed in the longitudinal study design [23]. The GEE model assesses the change in the average response for every 1-unit increase in a covariate across the population and can predict the coefficients of time-dependent variables in contrast to the fixed effects model [24]. Subgroup analyses were performed to investigate whether the annual amount of pension benefit was associated with depressive symptoms by using the GEE model. Furthermore, this study assessed the relationship between the proportion of public pension income in gross income and depressive symptoms. Finally, stratified analysis was performed by stratifying low- and high-income households by defining less than 60% of the equalized median income as low-income households [25]. All calculated *P* values were 2‐sided; *P* values <.05 were considered significant. All analyses were performed using SAS software (version 9.4; SAS Institute). This study follows the STROBE (Strengthening the Reporting of Observational Studies in Epidemiology) guidelines for observational studies [26].

### Ethics Approval

This study was approved by the institutional review board of Severance Hospital at Yonsei University College of Medicine (approval 4-2021-0310). Informed consent was waived by the institutional review board, as the data of KoWePS are open to the public and do not contain any personally identifiable information.

## Results

### Characteristics of the Study Participants

The study participants were more likely to be older than the excluded individuals, live with their family, have higher incomes, and be unemployed (Table S1 of Multimedia Appendix 1). Table 1 describes the participants’ general characteristics in the baseline year (2014). A total of 4541 participants were included, and the mean CESD-11 score was 6.13 (SD 5.41); 3128 (68.9%) participants did not receive public pension, 1225 (26.9%) received national pension, and 188 (4.1%) received specific corporate pension. Their mean CESD-11 scores were 6.72 (SD 5.55), 5.03 (SD 4.89), and 3.61 (SD 4.42), respectively. Table S2 of Multimedia Appendix 2 demonstrates the demographic characteristics of the study participants in 2014 according to pension type. The rate of not receiving public pensions among women was higher than that among men and among those aged 80 years or older. Furthermore, the lower the level of education and the lower the income, the higher was the rate of not being enrolled in public pensions.

Table 2 presents the results of the GEE model, which shows that the scores of CESD-11 were lower among those who received public pensions. After adjusting all covariates, including income-related factors, individuals receiving public pension, regardless of pension type, tended to have lower CESD-11 scores than those who did not receive public pension (national: β=–.734; *P*<.001; specific: β=–.775; *P*=.02). The results also showed that a higher realized property income was correlated with lower CESD-11 scores (β=–.283; *P*<.001). However, public or private transfer income were not associated with depressive symptoms.

Table 3 shows the results of the GEE model for the subgroup analyses of the associations among amount of pension benefit, proportion of public pension income, and depressive symptoms. After adjusting for all the covariates, older adults who received more than the fourth quartile among the specific corporate pension recipients reported the lowest depression score (β=–1.221; *P*=.001). For both national pension and specific corporate pension recipients, the CESD-11 scores tended to decrease as the amount of pension benefits increased. The CESD-11 score was significantly lowered when the proportion of national pension income over gross income was 75% or less (*P*<.001), but no statistical significance was found when it exceeded 75%. Meanwhile, when the proportion of specific corporate pension income over gross income exceeded 25%, the depression score decreased significantly (*P*<.05).

Table 4 shows the results of the analyses, which present the associations of public pension with depressive symptoms stratified by household income. In low-income households with less than 60% of equalized gross income, there was a clear relationship among public pension benefits, amount of benefits, and lower CESD-11 scores. Moreover, the CESD-11 score was significantly lowered even if the proportion of national pension income over gross income exceeded 75% (β=–2.783; *P*<.001).

**Table 1:** General characteristics of the study participants (2014 baseline year) (N=4541).

Variable		Values, n (%)	CESD-11^a^ score, mean (SD)^b^	*P* value	
**Pension type**					<.001
	No	3128 (68.9)	6.72 (5.55)		
	National pension	1225 (26.9)	5.03 (4.89)		
	Specific corporate pension	188 (4.1)	3.61 (4.42)		
**Sex**					<.001
	Male	1724 (38)	4.88 (4.97)		
	Female	2817 (62)	6.90 (5.53)		
**Age (years)**					<.001
	65-69	1066 (22.9)	4.92 (4.95)		
	70-74	1449 (32.1)	5.77 (5.22)		
	75-79	1181 (26.2)	6.47 (5.33)		
	≥80	845 (18.7)	7.82 (5.93)		
**Region**					<.001
	Metropolitan	1619 (35.7)	5.91 (5.40)		
	Urban	2771 (61)	6.32 (5.45)		
	Rural	151 (3.3)	5.15 (4.83)		
**Marital status**					<.001
	Married	2668 (58.8)	5.16 (5.06)		
	Divorced, widowed, separated, or never married	1873 (41.2)	7.52 (5.60)		
**Number of household members**					<.001
	1	1362 (30)	7.80 (5.59)		
	2	2380 (52.4)	5.40 (5.21)		
	≥3	799 (17.6)	5.49 (5.09)		
**Household income**					<.001
	High	1138 (25.1)	4.38 (4.55)		
	Upper-middle	1131 (24.9)	5.50 (5.26)		
	Lower-middle	1133 (25)	6.55 (5.57)		
	Low	1139 (25.1)	8.10 (5.51)		
**Highest level of education**					<.001
	College and higher	3725 (82)	6.50 (5.49)		
	High school	565 (12.4)	4.54 (4.75)		
	Middle school or lower	251 (5.5)	4.35 (4.59)		
**Employment type**					<.001
	Wage workers	495 (10.9)	4.87 (4.74)		
	Self-employed	816 (18)	4.94 (4.85)		
	Not employed	3230 (71.1)	6.63 (5.57)		
**Alcohol consumption**					<.001
	No	3374 (74.3)	6.67 (5.57)		
	~Once/week	566 (12.5)	4.55 (4.50)		
	>Once/week	601 (13.2)	4.63 (4.71)		
**Smoking status**					.40
	Nonsmoker	4054 (89.3)	6.11 (5.41)		
	Current smoker	487 (10.7)	6.29 (5.47)		
**Chronic diseases**					<.001
	No	447 (9.8)	4.03 (4.51)		
	Yes	4094 (90.2)	6.36 (5.45)		
Realized property income^c^		4541 (100)	1.59 (4.70)	<.001	
Public transfer income^d^		4541 (100)	1.10 (0.71)	<.001	
Private transfer income^e^		4541 (100)	4.22 (5.27)	<.001	
Household debt		4541 (100)	7.10 (29.99)	.26	

^a^CESD-11: 11-item Center for Epidemiologic Studies Depression (scale).

^b^Total mean (SD) score of all participants was 6.13 (5.41).

^c^Including interest income and rental income. The mean income was considered in US $1000.

^d^Including basic old age pension. The mean income was considered in US $1000.

^e^Including subsidy from children and personal pension. The mean income was considered in US $1000.

**Table 2:** Results of the generalized estimating equation analysis of factors associated with depressive symptoms.

Variables		CESD-11^a^ score, β (SE)	*P* value
**Pension type**			
	No	Reference	Reference
	National pension	–.734 (.110)	<.001
	Specific corporate pension	–.775 (.242)	.02
**Sex**			
	Male	Reference	Reference
	Female	.543 (.134)	<.001
**Age (years)**			
	65-69	Reference	Reference
	70-74	.033 (.100)	.74
	75-79	.259 (.122)	.03
	≥80	.779 (.144)	<.001
**Region**			
	Metropolitan	Reference	Reference
	Urban	.129 (.109)	.24
	Rural	–.422 (.265)	.11
**Marital status**			
	Married	Reference	Reference
	Divorced, widowed, separated, or never married	.654 (.169)	<.001
**Number of household members**			
	1	Reference	Reference
	2	–.415 (.184)	.02
	≥3	–.195 (.216)	.37
**Household income**			
	High	Reference	Reference
	Upper middle	.656 (.109)	<.001
	Lower middle	1.055 (.127)	<.001
	Low	1.501 (.163)	<.001
**Highest level of education**			
	College and higher	Reference	Reference
	High school	.099 (.238)	.68
	Middle school or lower	.561 (.227)	.01
**Employment type**			
	Wage workers	Reference	Reference
	Self-employed	.294 (.142)	.04
	Not employed	.899 (.110)	<.001
**Alcohol consumption**			
	No	Reference	Reference
	~Once/week	–.536 (.099)	<.001
	>Once/week	–.614 (.117)	<.001
**Smoking status**			
	Nonsmoker	Reference	Reference
	Current smoker	.749 (.161)	<.001
**Chronic diseases**			
	No	Reference	Reference
	Yes	1.015 (.110)	<.001
Realized property income^b^ per US $10,000/year higher		–.283 (.074)	<.001
Public transfer income^c^ (including basic old age pension) per US $10,000/year higher		–.474 (.283)	.09
Private transfer income^d^ per US $10,000/year higher		.059 (.074)	.42
Household debt per US $10,000/year higher		.021 (.005)	<.001

^a^CESD-11: 11-item Center for Epidemiologic Studies Depression (scale).

^b^Including interest income and rental income. If the participant’s realized property income increases by US $10,000 per year, the estimated 11-item Center for Epidemiologic Studies Depression (scale) score will decrease by .283 points.

^c^Including basic old age pension.

^d^Including subsidy from children and personal pension.

**Table 3:** Results of the generalized estimating equation model for the subgroup analyzes of the associations among amount of pension benefit, proportion of public pension income, and depressive symptoms.

Variables				CESD-11^a^ score, β (SE)		*P* value^b^
**Amount of pension benefit**						
	No			Reference		Reference
	**National pension**					
		~25% (US $1440/year)	–.676 (.154)		<.001	
		~75% (US $2790/year)	–.703 (.124)		<.001	
		~100% (US $29,400/year)	–.979 (.175)		<.001	
	**Specific corporate pension**					
		~25% (US $16,800/year)	–.548 (.380)		.15	
		~75% (US $27,600/year)	–.834 (.291)		.004	
		~100% (US $44,880/year)	–1.221 (.375)		.001	
**Proportion of pension income**						
	No			Reference		Reference
	**National pension**					
		0<pension income/gross income≤0.25	–.665 (.112)		<.001	
		0.25<pension income/gross income≤0.75	–1.220 (.208)		<.001	
		0.75<pension income/gross income≤1.0	–.992 (.881)		.26	
	**Specific corporate pension**					
		0<pension income/gross income≤0.25	–.179 (.795)		.82	
		0.25<pension income/gross income≤0.75	–.746 (.266)		.005	
		0.75<pension income/gross income≤1.0	–.929 (.305)		.002	

^a^CESD-11: 11-item Center for Epidemiologic Studies Depression (scale).

^b^Adjusted for all covariates.

**Table 4:** Results of generalized estimating equation model for subgroup analyzes of association of amount of pension benefit and proportion of pension in gross income with regard to pension type with depressive symptoms stratified by household income.

Variables							CESD-11^a^ score, β (SE)			*P* value^b^
**Amount of pension benefit**										
	**High-income households (** **≥** **60%)**									
			No			Reference			Reference	
			**National pension**							
				~25% (US $1440/year)	–.694 (.215)			.001		
				~75% (US $2790/year)	–.449 (.165)			.007		
				~100% (US $29,400/year)	–.575 (.211)			.006		
			**Specific corporate pension**							
				~25% (US $16,800/year)	–.426 (.480)			.38		
				~75% (US $27,600/year)	–.432 (.312)			.17		
				~100% (US $44,880/year)	–.848 (.387)			.03		
	**Low-income households (<60%)**									
			No			Reference			Reference	
			**National pension**							
				~25% (US $1440/year)	–.663 (.195)			<.001		
				~75% (US $2790/year)	–.861 (.162)			<.001		
				~100% (US $29,400/year)	–1.472 (.266)			<.001		
			**Specific corporate pension**							
				~25% (US $16,800/year)	–.782 (.531)			.14		
				~75% (US $27,600/year)	–3.646 (.642)			<.001		
				~100% (US $44,880/year)	Undetectable			N/A^c^		
**Proportion of pension income**										
	**High-income households (** **≥** **60%)**									
		No				Reference			Reference	
		**National pension**								
				0<pension income/gross income≤0.25	–.532 (.149)			<.001		
				0.25<pension income/gross income≤0.75	–.704 (.389)			.07		
				0.75<pension income/gross income≤1.0	.357 (1.429)			.80		
		**Specific corporate pension**								
				0<pension income/gross income≤0.25	–.318 (.897)			.72		
				0.25<pension income/gross income≤0.75	–.686 (.294)			.02		
				0.75<pension income/gross income≤1.0	–.581 (.353)			.10		
	**Low-income households (<60%)**									
			No			Reference			Reference	
			**National pension**							
				0<pension income/gross income≤0.25	–.625 (.150)			<.001		
				0.25<pension income/gross income≤0.75	–1.254 (.242)			<.001		
				0.75<pension income/gross income≤1.0	–2.783 (.835)			<.001		
			**Specific corporate pension**							
				0<pension income/gross income≤0.25	.188 (1.400)			.89		
				0.25<pension income/gross income≤0.75	–.443 (.743)			.55		
				0.75<pension income/gross income≤1.0	–1.599 (.594)			.007		

^a^CESD-11: 11-item Center for Epidemiologic Studies Depression (scale).

^b^Adjusted for all covariates.

^c^N/A: not applicable.

## Discussion

### Principal Results

This longitudinal study investigates whether receiving public pension, which plays a major role in the old age income security policy in Korea, was associated with depressive symptoms in older adults. In particular, we distinguished between national and specific corporate pensions, which are the 2 pillars of public pension in Korea and evaluated whether the 2 types of pensions could improve depressive symptoms. We found that the depression scores decreased as the amount of benefits increased for both public pensions. Regardless of the type, the association between public pension benefits and decreased depressive symptoms was pronounced in low-income households. The results of our study imply that financial resources provided through public pensions can improve the mental health of older people.

Conflicting findings have been reported regarding the relationship between pension income and health [27,28]. However, care must be taken when interpreting the relationship between pensions and health, as each country has different forms and types of pensions. Many European countries have studied the association between disability pensions and health outcomes [29]. Research in Europe has shown that receiving a disability pension can have a more negative impact on mental health than not receiving it. It is presumed that this phenomenon is due to the psychosocial stress experienced by beneficiaries in obtaining eligibility for disability pension. Additionally, claims stigma can have a negative impact on mental health [30].

Contrary to the findings in the aforementioned reports, our findings show a positive relationship between public pension as a means of maintaining income in old age and mental health. Recently, many studies in China have found that China’s new rural pension scheme reduces depressive symptoms and lowers the prevalence of depression [9,31,32]. The findings of a Japanese study on the relationship between contribution-based public pensions and happiness were also similar to our study results [19]. Furthermore, the results of a study analyzing the relationship between aged pension schemes and mental health conducted in New Zealand reported that material hardship can cause health inequities, including mental health [33]. These studies suggest that older adults can benefit their mental health by reducing labor supply and spending more leisure time with friends and family through the pension income. Additionally, pensions can reduce depressive symptoms by strengthening the financial capacity of beneficiaries and their families. However, there are limitations in that the existing studies [9,31,32] examined the effects of pension systems that were implemented in rural areas or had cross-sectional research designs. Under these circumstances, we evaluated the association between contributory public pensions and mental health after adjusting for each individual’s pension income type, considering the multilayered Korean pension system.

In this study, among older adults without a history of diagnosed depression, those receiving contribution-based public pensions, regardless of pension type, were less likely to experience depressive symptoms than those who did not receive public pensions. Additionally, in subgroup analyses, we observed that the CESD-11 scores tended to decrease as the amount of pension benefits increased for both national pension and specific corporate pension recipients. These results imply that the policy of guaranteeing old age income through public pensions was associated with a lower depressive score and that the larger the amount of public pension benefits, the better was the mental health.

Several studies have evaluated that pensions could affect mental health through the following pathways: first, through changes in lifestyle habits such as independent living and leisure time; second, by enabling healthy life choices such as with nutrition and medical treatment; and third, by reducing economic stress [9,34,35]. In addition to the 3 pathways mentioned above, the relation between contribution-based public pension receipt and alleviation of depressive symptoms can be explained by the following mechanisms. In the case of a contribution-based public pension, the result of one’s labor is returned later. Therefore, there is a possibility that even a small amount of money may make one feel satisfied, believing that the pension is a reward for one’s effort [36]. In particular, given the hedonic adaptation hypothesis, according to which the pleasure of economic benefits gradually disappears after receiving a noncontributory pension [37], the contribution-based pension has a different mechanism in relieving depressive symptoms in that it reflects the results of previous labor. Another possibility is that public pension recipients were economically active and stable when they were young, since they earned income from work or self-employment for at least 10 years or more, contributing to the pension. This may have lowered their likelihood of experiencing depressive symptoms. In addition, the experience of working long-term at a company or being self-employed to contribute to public pensions may have affected their social networks, which may ultimately influence their mental health [38]. However, no association with lower depressive symptoms was observed when the pension amount among national pension recipients was 75% or more of the gross income, although the CESD-11 score tended to decrease. Considering our research showing that the top 25% of national pension recipients receive only US $2790 per year (US $232.50 per month), there is a possible limitation to improving depressive symptoms merely if older adults receive public pension, requiring them to live with fewer pension benefits. This phenomenon is in line with the results of this study and previous studies, which indicate that receiving a low amount of noncontributing basic old age pension was not associated with decreased depressive symptoms [39]. Nevertheless, among low-income people, public pensions were associated with low CESD-11 scores, even for low amounts. These results suggest that for low-income people, even a small amount of national pension can improve their depressive symptoms and that the level of income should be considered when formulating a pension policy.

In our study, even for a specific corporate pensioner whose pension benefit amount was higher than that of the national pensioner, pension receipt was not associated with alleviation of depressive symptoms for individuals whose pension amount was in the bottom 25% or less than 25% of the gross income among specific corporate pensioners. Depressive symptoms may not decrease for those with low specific corporate pension benefits because they must earn income through other means. In Korea, the corporate pension amount is higher than the national pension amount. Thus, specific corporate pension beneficiaries do not have to worry about their postretirement income. Our results suggest that it is necessary to consider the types of public pensions and their implications for beneficiaries in addition to the pension receipt or amount, when studying the effects of public pensions on mental health.

The state’s finances are limited; thus, tax-based noncontribution pensions cannot be increased indefinitely. Moreover, it is almost impossible to expand noncontributory pensions, especially in low-income countries, which are forced to spend a considerable share of the state’s finances on economic development. Therefore, the few financial policies that can be used to improve the mental health of older adults are contribution-based pensions. The objective of the public pension policy is to guarantee a certain amount of benefits to individuals so that they do not experience economic difficulties when they become older. This can be achieved, for example, by increasing contributions or by extending the period of contributions when people are young.

Taken together, our findings may provide several important implications not only for the Korean society but also for countries planning and revising pension policies. First, our findings justify a wide range of policy interventions that promote public health based on retirement income security policies such as public pensions. Second, these findings suggest that mental health can be effectively improved with economic resources. Third, the best way to use economic resources to improve mental health is to ensure an income that is economically sufficient. However, creating this condition without contributions can be difficult. Fourth, for low-income countries that cannot afford to preserve older adults’ income, a contribution-based public pension system may be an important policy in order to maintain the mental health of older adults after retirement.

### Limitations

This study has several limitations. First, the exact causal relationship between public pension benefit and depressive symptoms could not be confirmed, although this study used longitudinal data with repeated observations at the individual level over a specific period [40]. Second, residual confounding may exist because of income-related factors. Finally, since the economic and cultural basis that determines the pension policy and the direction of policy in each country may differ, direct application of the results of this study may be limited [41]. Longitudinal studies in more countries and based on larger numbers of public pension recipients are needed to confirm causal relationships.

### Conclusions

This study evaluates both the national and specific corporate pensions, which are the 2 pillars of public pensions, and their association with lower prevalence of depressive symptoms in Korea. The depression scores decreased as the amount of benefits increased for both public pensions. However, for beneficiaries of specific corporate pensions with lower benefits, the association with depressive symptoms was absent. Regardless of the pension type, the association between public pension benefits and decreased depressive symptoms was pronounced in low-income households. For low-income countries that cannot afford to preserve older adults’ income, the contribution-based public pension system may be an important policy to maintain the mental health of older adults after retirement.

## Appendix

Multimedia Appendix 1 Characteristics of the study samples and excluded participants owing to missing data (2014 baseline year).

Multimedia Appendix 2 General characteristics of the study participants according to pension type (2014 baseline year).

## Abbreviations

CESD-11: 11-item Center for Epidemiologic Studies Depression (scale)
GEE: generalized estimating equation
KoWePS: Korea Welfare Panel Study
STROBE: Strengthening the Reporting of Observational Studies in Epidemiology

## Data Availability

All the Korea Welfare Panel Study data used in this study are available to the public and can be seen in the official website of the Korea Welfare Panel Study.
