# Supplementary material for: Association of the Type of Public Pension With Mental Health Among South Korean Older Adults: Longitudinal Observational Study
Source: JMIR Public Health Surveill. 2024 May 2;10:e49129. doi: 10.2196/49129 (PMC11099812; doi:10.2196/49129)
Supplement: Multimedia Appendix 1 [file publichealth_v10i1e49129_app1.docx]

| **Table S1. Characteristics of study samples and excluded subjects owing to missing data (2014 baseline year)** | | | | | | | |
| --- | --- | --- | --- | --- | --- | --- | --- |
| **Variables** |  | **Study sample  (n=4,541)** | |  | **Excluded sample  (n=254)** | | ***P-*  value ^a^** |
|  |  | **N** | **%** |  | **N** | **%** |  |
| **Pension type** |  |  |  |  |  |  | .07 |
| No |  | 3,128 | 68.9 |  | 68 | 78.2 |  |
| Public pension |  | 1,225 | 27.0 |  | 14 | 16.1 |  |
| Specific corporate pension |  | 188 | 4.1 |  | 5 | 5.7 |  |
| **Sex** |  |  |  |  |  |  | <.001 |
| Male |  | 1,724 | 38.0 |  | 126 | 49.6 |  |
| Female |  | 2,817 | 62.0 |  | 128 | 50.4 |  |
| **Age** |  |  |  |  |  |  | <.001 |
| 65-69 |  | 1,066 | 23.5 |  | 39 | 15.3 |  |
| 70-74 |  | 1,449 | 31.9 |  | 61 | 24.0 |  |
| 75-79 |  | 1,181 | 26.0 |  | 53 | 20.9 |  |
| 80- |  | 845 | 18.6 |  | 101 | 39.8 |  |
| **Region** |  |  |  |  |  |  | .37 |
| Metropolitan |  | 1,619 | 35.7 |  | 81 | 31.9 |  |
| Urban |  | 2,771 | 61.0 |  | 162 | 63.8 |  |
| Rural |  | 151 | 3.3 |  | 11 | 4.3 |  |
| **Marital status** |  |  |  |  |  |  | .18 |
| Married |  | 2,668 | 58.8 |  | 160 | 63.0 |  |
| Divorced, widowed, separated  or never married |  | 1,873 | 41.2 |  | 94 | 37.0 |  |
| **Number of household members** |  |  |  |  |  |  | <.001 |
| 1 |  | 1,362 | 30.0 |  | 3 | 1.2 |  |
| 2 |  | 2,380 | 52.4 |  | 160 | 63.0 |  |
| ≥3 |  | 799 | 17.6 |  | 91 | 35.8 |  |
| **Family income level** |  |  |  |  |  |  | <.001 |
| High |  | 1,138 | 25.1 |  | 103 | 40.5 |  |
| Upper middle |  | 1,131 | 24.9 |  | 70 | 27.6 |  |
| Lower middle |  | 1,133 | 25.0 |  | 53 | 20.9 |  |
| Low |  | 1,139 | 25.1 |  | 28 | 11.0 |  |
| **Highest level of education** |  |  |  |  |  |  | .52 |
| Middle school or under |  | 3,725 | 82.0 |  | 200 | 80.3 |  |
| High school |  | 565 | 12.4 |  | 31 | 12.4 |  |
| College and above |  | 251 | 5.5 |  | 18 | 7.2 |  |
| **Employment type** |  |  |  |  |  |  | <.001 |
| Wage workers |  | 495 | 10.9 |  | 4 | 1.6 |  |
| Self-employed |  | 816 | 18.0 |  | 7 | 2.8 |  |
| Nonemployee |  | 3,230 | 71.1 |  | 243 | 95.6 |  |
| **Alcohol consumption** |  |  |  |  |  |  | .01 |
| No |  | 3,374 | 74.3 |  | 77 | 88.5 |  |
| ~ Once/week |  | 566 | 12.5 |  | 6 | 6.9 |  |
| > Once/week |  | 601 | 13.2 |  | 4 | 4.6 |  |
| **Smoking status** |  |  |  |  |  |  | .25 |
| Non-smoker |  | 4,054 | 89.3 |  | 81 | 93.1 |  |
| Current smoker |  | 487 | 10.7 |  | 6 | 6.9 |  |
| **Chronic diseases** |  |  |  |  |  |  | .21 |
| No |  | 447 | 9.8 |  | 19 | 7.5 |  |
| Yes |  | 4,094 | 90.2 |  | 235 | 95.5 |  |
| **Realized property income ^b^** |  |  |  |  |  |  |  |
| Mean (1,000 dollar), SD |  | 1.59 | 4.70 |  | 1.70 | 5.80 | .77 |
| **Public transfer income ^c^** |  |  |  |  |  |  |  |
| Mean (1,000 dollar), SD |  | 1.10 | 0.71 |  | 1.27 | 0.75 | .001 |
| **Private transfer income ^d^** |  |  |  |  |  |  |  |
| Mean (1,000 dollar), SD |  | 4.22 | 5.27 |  | 4.74 | 8.32 | .32 |
| **Household debt** |  |  |  |  |  |  |  |
| Mean (1,000 dollar), SD |  | 7.10 | 29.99 |  | 12.04 | 41.83 | .07 |
| Total |  | 4,541 | 100.0 |  | 254 | 100.00 |  |
| ^a^ *P*-values were obtained by Chi-square tests or T-test | | | | | | | |
| ^b^ Including interest income and rental income | | | | | | | |
| ^c^ Including basic old-age pension | | | | | | | |
| ^d^ Including subsidy from children and personal pension | | | | | | | |
| Abbreviations: SD, standard deviation | | | | | | | |
