# Supplementary material for: Association of the Type of Public Pension With Mental Health Among South Korean Older Adults: Longitudinal Observational Study
Source: JMIR Public Health Surveill. 2024 May 2;10:e49129. doi: 10.2196/49129 (PMC11099812; doi:10.2196/49129)
Supplement: Multimedia Appendix 2 [file publichealth_v10i1e49129_app2.docx]

| **Table S2. General characteristics of study subjects according to pension type (2014 baseline year)** | | | | | | | | | | | | | | | | | | |  |
| --- | --- | --- | --- | --- | --- | --- | --- | --- | --- | --- | --- | --- | --- | --- | --- | --- | --- | --- | --- |
| **Variables** |  | **Pension type** | | | | | | | | | | | | | | | | | |
|  |  | **Total** | | | | **No** | | | | **National pension** | | | | **Specific corporate pension** | | | | ***P -value*** | |
|  |  | **N** | | **%** | | **N** | | **%** | | **N** | | **%** | | **N** | | **%** | |  |  |
| **Total** |  | **4,541** | | **100** | | **3,128** | | **68.9** | | **1,225** | | **27.0** | | **188** | | **4.1** | |  | |
| **Sex** |  |  | |  | |  | |  | |  | |  | |  | |  | | <.001 | |
| Male |  | 1,724 | | 38.2 | | 829 | | 48.1 | | 744 | | 43.2 | | 151 | | 8.8 | |  | |
| Female |  | 2,817 | | 62.4 | | 2,299 | | 81.6 | | 481 | | 17.1 | | 37 | | 1.3 | |  | |
| **Age** |  |  | |  | |  | |  | |  | |  | |  | |  | | <.001 | |
| 65-69 |  | 1,066 | | 23.6 | | 554 | | 52.0 | | 457 | | 42.9 | | 55 | | 5.2 | |  | |
| 70-74 |  | 1,449 | | 32.1 | | 897 | | 61.9 | | 494 | | 34.1 | | 58 | | 4.0 | |  | |
| 75-79 |  | 1,181 | | 26.2 | | 911 | | 77.1 | | 216 | | 18.3 | | 54 | | 4.6 | |  | |
| 80- |  | 845 | | 18.7 | | 766 | | 90.7 | | 58 | | 6.9 | | 21 | | 2.5 | |  | |
| **Region** |  |  | |  | |  | |  | |  | |  | |  | |  | | .001 | |
| Metropolitan |  | 1,619 | | 35.9 | | 1,130 | | 69.8 | | 404 | | 25.0 | | 85 | | 5.3 | |  | |
| Urban |  | 2,771 | | 61.4 | | 1,900 | | 68.6 | | 772 | | 27.9 | | 99 | | 3.6 | |  | |
| Rural |  | 151 | | 3.3 | | 98 | | 64.9 | | 49 | | 32.5 | | 4 | | 2.6 | |  | |
| **Marital status** |  |  | |  | |  | |  | |  | |  | |  | |  | | <.001 | |
| Married |  | 2,668 | | 59.1 | | 1,726 | | 64.7 | | 796 | | 29.8 | | 146 | | 5.5 | |  | |
| Divorced, widowed, separated  or never married |  | 1,873 | | 41.5 | | 1,402 | | 74.9 | | 429 | | 22.9 | | 42 | | 2.2 | |  | |
| **Number of household members** |  |  | |  | |  | |  | |  | |  | |  | |  | | <.001 | |
| 1 |  | 1,362 | | 30.2 | | 1,006 | | 73.9 | | 323 | | 23.7 | | 33 | | 2.4 | |  | |
| 2 |  | 2,380 | | 52.7 | | 1,576 | | 66.2 | | 683 | | 28.7 | | 121 | | 5.1 | |  | |
| ≥3 |  | 799 | | 17.7 | | 546 | | 68.3 | | 219 | | 27.4 | | 34 | | 4.3 | |  | |
| **Household income** |  |  | |  | |  | |  | |  | |  | |  | |  | | <0.01 | |
| High |  | 1,138 | | 25.2 | | 651.0 | | 57.2 | | 350 | | 30.8 | | 137 | | 12.0 | |  | |
| Upper middle |  | 1,131 | | 25.1 | | 702.0 | | 62.1 | | 383 | | 33.9 | | 46 | | 4.1 | |  | |
| Lower middle |  | 1,133 | | 25.1 | | 829.0 | | 73.2 | | 299 | | 26.4 | | 5 | | 0.4 | |  | |
| Low |  | 1,139 | | 25.2 | | 946.0 | | 83.1 | | 193 | | 16.9 | | 0 | | 0.0 | |  | |
| **Highest level of education** |  |  | |  | |  | |  | |  | |  | |  | |  | | <.001 | |
| College and above |  | 251 | | 5.6 | | 117 | | 46.6 | | 64 | | 25.5 | | 70 | | 27.9 | |  | |
| High school |  | 565 | | 12.5 | | 273 | | 48.3 | | 220 | | 38.9 | | 72 | | 12.7 | |  | |
| Middle school or under |  | 3,725 | | 82.5 | | 2,738 | | 73.5 | | 941 | | 25.3 | | 46 | | 1.2 | |  | |
| **Employment type** |  |  | |  | |  | |  | |  | |  | |  | |  | | <.001 | |
| Wage workers |  | 495 | | 11.0 | | 279 | | 56.4 | | 202 | | 40.8 | | 14 | | 2.8 | |  | |
| Self-employed |  | 816 | | 18.1 | | 392 | | 48.0 | | 393 | | 48.2 | | 31 | | 3.8 | |  | |
| Nonemployee |  | 3,230 | | 71.6 | | 2,457 | | 76.1 | | 630 | | 19.5 | | 143 | | 4.4 | |  | |
| **Alcohol consumption** |  |  | |  | |  | |  | |  | |  | |  | |  | | <.001 | |
| No |  | 3,374 | | 74.7 | | 2,518 | | 74.6 | | 750 | | 22.2 | | 106 | | 3.1 | |  | |
| ~ Once/week |  | 566 | | 12.5 | | 318 | | 56.2 | | 206 | | 36.4 | | 42 | | 7.4 | |  | |
| > Once/week |  | 601 | | 13.3 | | 292 | | 48.6 | | 269 | | 44.8 | | 40 | | 6.7 | |  | |
| **Smoking status** |  |  | |  | |  | |  | |  | |  | |  | |  | | <.001 | |
| Non-smoker |  | 4,054 | | 89.8 | | 2,838 | | 70.0 | | 1,051 | | 25.9 | | 165 | | 4.1 | |  | |
| Current smoker |  | 487 | | 10.8 | | 290 | | 59.5 | | 174 | | 35.7 | | 23 | | 4.7 | |  | |
| **Chronic diseases** |  |  | |  | |  | |  | |  | |  | |  | |  | | <.001 | |
| No |  | 447 | | 9.9 | | 279 | | 62.4 | | 138 | | 30.9 | | 30 | | 6.7 | |  | |
| Yes |  | 4,094 | | 90.7 | | 2,849 | | 69.6 | | 1,087 | | 26.6 | | 158 | | 3.9 | |  | |
| **Realized property income ^a^**  **(Mean dollar, SD)** | 4,541 | | 100 | | 1,415 | | 4,580 | | 1,878 | | 5,004 | | 2,681 | | 4,480 | | <.001 | |  |
| **Public transfer income ^b^**  **(Mean dollar, SD)** |  | 4,541 | | 100 | | 1,209 | | 655 | | 984 | | 736 | | 44 | | 235 | | <.001 | |
| **Private transfer income ^c^**  **(Mean dollar, SD)** | 4,541 | | 100 | | 4364 | | 5544 | | 3996 | | 4686 | | 3176 | | 3877 | | .002 | |  |
| **Household debt**  **(Mean dollar, SD)** |  | 4,541 | | 100 | | 6,791 | | 30,747 | | 7,902 | | 28,750 | | 7,111 | | 24,891 | | .51 | |
| ^a^ Including interest income and rental income | | | | | | | | | | | | | | | | | | |  |
| ^b^ Including basic old-age pension | | | | | | | | | | | | | | | | | | |  |
| ^c^ Including subsidy from children and personal pension | | | | | | | | | | | | | | | | | | |  |
| P-values were calculated by the chi-squared test for categorical variables and the ANOVA test for continuous variables. | | | | | | | | | | | | | | | | | | |  |
